# Report of Deaths in the City of Buffalo, for the Month of July, 1862

**Published:** 1862-09

**Authors:** 


*Report of Deaths in the City of Buffalo, for the Month of July,* 1862:

Accident, 9; Accident by Drowning, 4; Apoplexy, Cerebral, 1; Concussion of Brain, 1; Cancer of the Stomach, 1; Cancer of the Womb, 1; Cholera Infantum, 8; Consumption, 14; Convulsions, 12; Cyanosis, 1; Debility, 2; Delirium Tremens, 2; Dentition, 1; Diabetes Mellitus, 1; Diarrhœa, 11; Disease of the Brain, 2; Disease of the Heart, 3; Diptheria, 2; Dropsy, General, 2; Epilepsy, 2; Erysipelas, 1; Fever, 1; Fever, Puerperal, 2; Fever, Scarlet, 18; Fever, Typhoid, 3; Hæmorrhage, 1; Inflammation of Bowels, 3; Inflammation of Brain, 1; Inflammation and Meninges, 4; Inflammation of the Lungs, 6; Inflammation of the Lungs, Typhoid, 1; Inflammation of the Womb, 1; Intemperance, 4; Kidneys, Bright's disease of, 1; Marasmus, 4; Measles, 6; Old Age, 5: Premature Birth, 2; Pyæmia, 1; Scrofula, 2; Suicide, 1; Tabes Mesenterica, 1; Unknown, 3;—Total, 152. Ages: 1 and 30 days, 11; 1 month and 6 months, 18; 6 months and 1 year, 12; 1 to 3 years, 24; 3 to 5 years, 9; 5 to 10 years, 11; 10 to 20 years, 11; 20 to 30 years 11; 30 to 40 years, 13; 40 to 50 years, 12; 50 to 60 years, 7; 60 to 70 years, 3; 70 to 80 years, 5; 80 to 90 years, 4; Stillborn, 4;—Total, 156. Sex: Males, 85; Females, 71. Color: White, Male, 1; White, Female, 70; Unknown, 3. Nativities: United States, 106; German States, 23; Ireland, 17; England, 4; France, 3; Scotland, 1; Poland, 1; Nova Scotia, 1. Parentage: American, 15; German, 77; Irish, 28; English, 5; Scotch, 5; French, 5; Holland, 1; Canada, 21; Nova Scotia, 1; Unknown, 18. Condition: Married, 25; Single, 104; Widows, 3; Widowers, 3; Unknown, 21. Locality: City at large, 136; Hospital of Sisters of Charity, 8; Buffalo General Hospital, 2; Catholic Foundling Asylum, 5.

